# Hepatic involvement as an initial manifestation of multiple myeloma: a case report with atypical imaging features

**DOI:** 10.3389/fonc.2025.1640981

**Published:** 2025-09-10

**Authors:** Zongsheng Pu, Lujiang Zhan, Yimi Zha, Yilin Li, Shilin Qiu, Yinfu He, Yanhong Yang, Zaihui Feng, Xingxing Zhu

**Affiliations:** ^1^ Department of Radiology, The Third People’s Hospital of Honghe Hani and Yi Autonomous Prefecture, Gejiu, Yunnan, China; ^2^ Department of Pathology, The Third People’s Hospital of Honghe Hani and Yi Autonomous Prefecture, Gejiu, Yunnan, China

**Keywords:** multiple myeloma, hepatic involvement, imaging, extramedullary myeloma, differential diagnosis

## Abstract

Hepatic involvement as the initial manifestation of multiple myeloma (MM) is exceedingly rare and presents significant diagnostic challenges due to its heterogeneous imaging features, which often mimic metastatic liver tumors. We report a case of a 62-year-old man presenting with right upper quadrant discomfort. Imaging revealed a hypoechoic lesion in segment II of the liver on ultrasound, a low-density area with mild enhancement on contrast-enhanced CT, and atypical MRI features including hyperintensity on T1- and T2-weighted images, mild diffusion restriction on diffusion-weighted imaging (DWI), and minimal enhancement post-Gd-DTPA administration. Histopathology demonstrated diffuse infiltration of atypical plasma cells consistent with plasmacytoma, supported by immunohistochemical positivity for CD138, CD38, MUM1, and CD56, and an elevated serum β_2_-microglobulin level, confirming the diagnosis of MM with hepatic involvement. This case underscores the variability of extramedullary myeloma (EM) imaging presentations in the liver and highlights the importance of including EM in the differential diagnosis of atypical hepatic lesions, especially when accompanied by osteolytic bone lesions or abnormal serum markers. Histopathological confirmation remains essential for definitive diagnosis and guiding treatment.

## Introduction

Multiple myeloma (MM) is a malignant clonal proliferation of plasma cells within the bone marrow, presenting with diverse clinical and radiological manifestations. When the disease extends beyond the bone marrow and invades soft tissues, it is termed extramedullary myeloma (EM) (1). The most common sites of EM involvement are the skin and muscles, accounting for approximately 24% of cases (2). Current evidence indicates that EM is associated with a poor prognosis, with outcomes worse than MM cases without soft tissue plasmacytomas (3, 4). Therefore, early diagnosis is crucial for the effective management of EM patients. The liver is among the potential sites of EM involvement; however, the non-specific imaging characteristics of hepatic EM often resemble other neoplastic or inflammatory lesions, increasing the risk of misdiagnosis or missed diagnosis. This poses significant challenges for clinical diagnosis and treatment decisions. Thus, a comprehensive understanding of the imaging features of hepatic EM is essential to improve detection and diagnostic accuracy. Herein, we report a rare case presenting initially as a hepatic mass, ultimately diagnosed as multiple myeloma with liver involvement. This case aims to enhance understanding of the radiological characteristics of hepatic EM.

## Case presentation

In March 2025, a 62-year-old man presented with discomfort in the right upper quadrant. Physical examination revealed mild tenderness in this area. Laboratory tests showed mildly elevated r-glutamyl transferase (r-GT), total protein (TP), globulin (GLO), and carbohydrate antigen CA - 125, while other liver function parameters, complete blood count, and tumor markers remained within normal limits. The patient exhibited no systemic symptoms such as jaundice, fever, or weight loss, and had no history of liver disease or malignancy.

**Figure 1:**
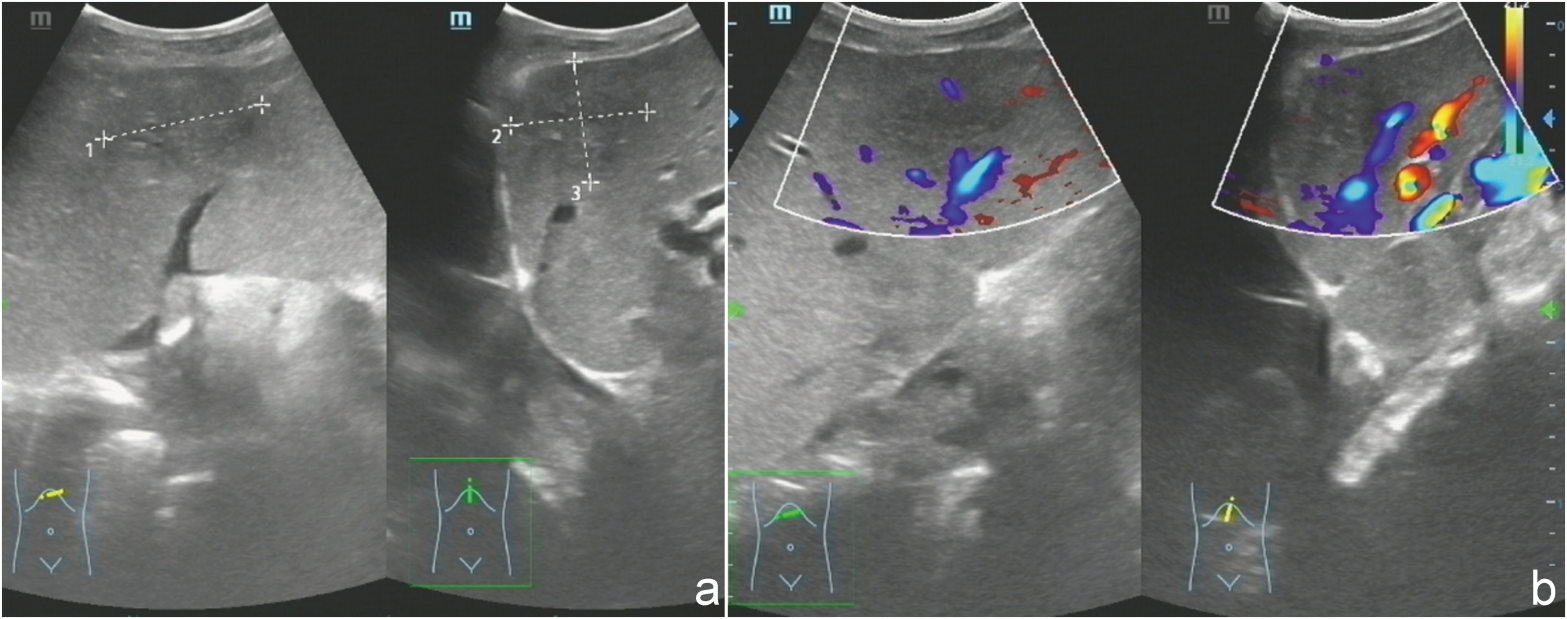
**(a)** Ultrasonography demonstrated a heterogeneous, patchy hypoechoic lesion with poorly defined margins located in segment S2 of the liver. **(b)** Color Doppler flow imaging (CDFI) revealed scattered intralesional and perilesional vascular signals.

**Figure 2:**
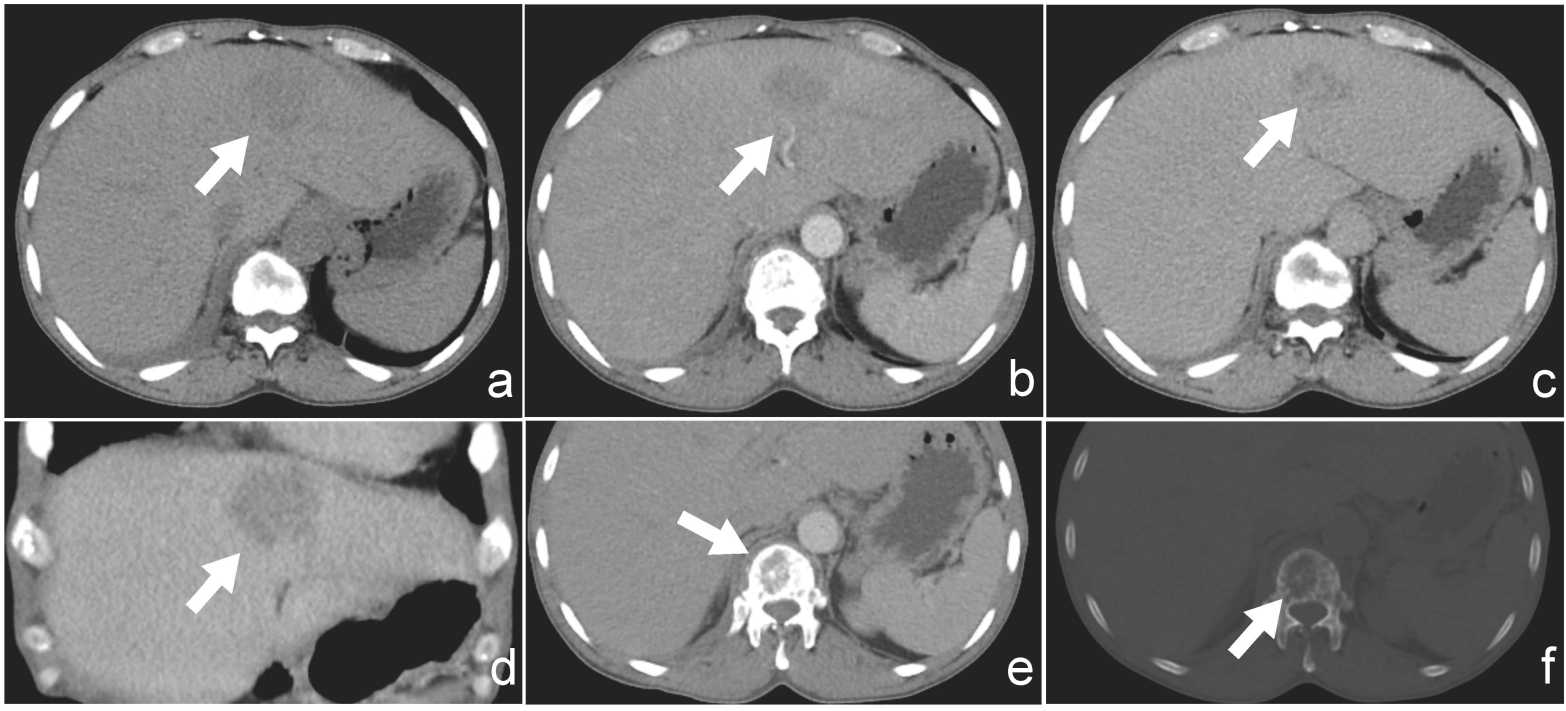
**(a)** Non-contrast CT image showing a hypodense lesion located in segment II of the liver. **(b)** Arterial phase contrast-enhanced CT showing mild peripheral enhancement of the lesion. **(c)** Delayed phase image demonstrates progressive enhancement of the lesion. **(d)** Coronal view of contrast-enhanced CT illustrating the lesion’s extent and enhancement pattern. **(e)** CT with soft-tissue windows shows no evidence of paravertebral or epidural soft-tissue involvement. **(f)** CT bone window reveals a lytic lesion involving the first lumbar vertebra (L1), without collapse or fracture.

**Figure 3:**
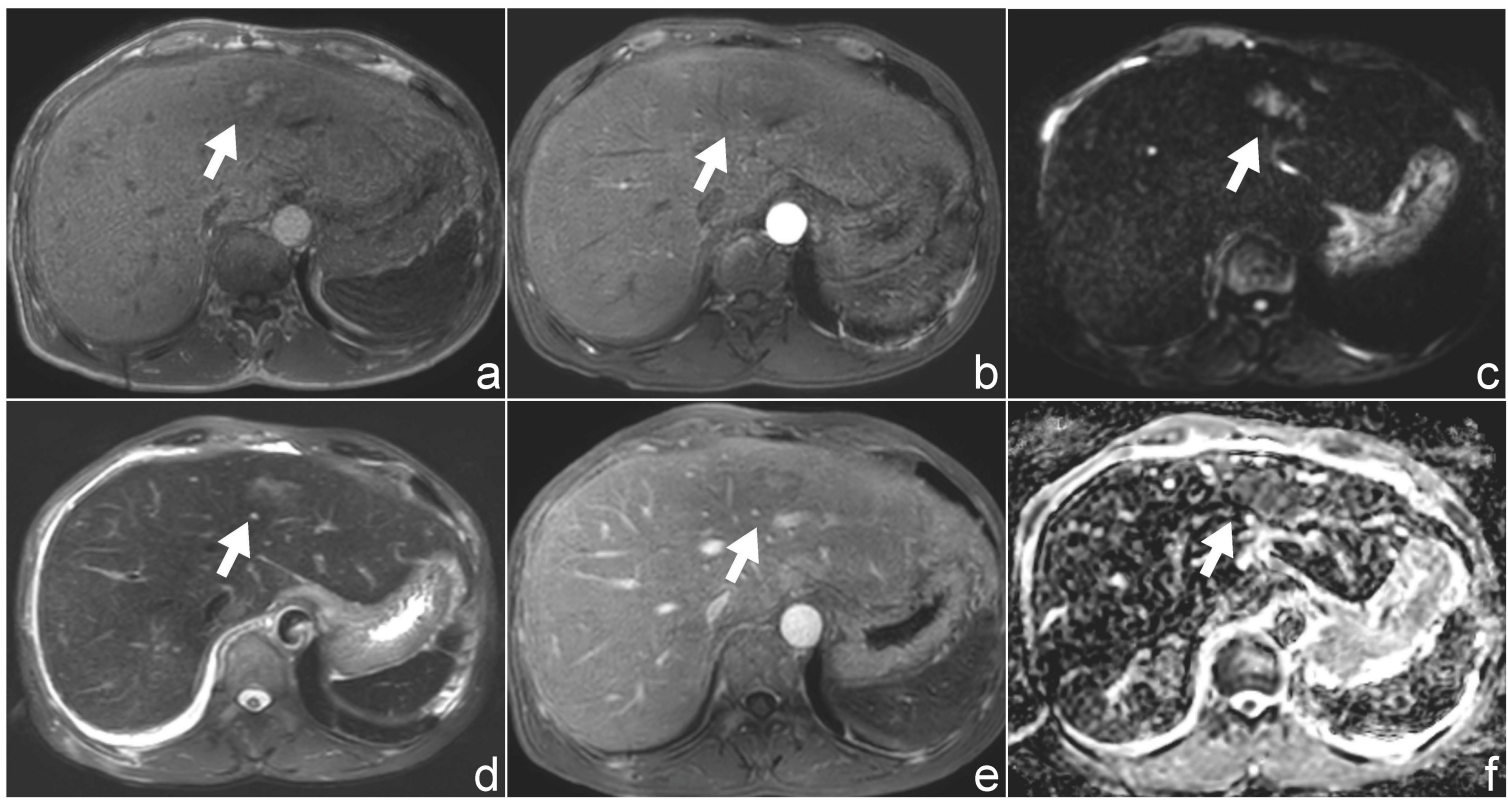
Magnetic resonance imaging (MRI) findings of the patient. **(a, d)** Axial T1-weighted **(a)** and T2-weighted **(d)** images demonstrate a hyperintense lesion in hepatic segment II. **(b, e)** Post-contrast T1-weighted images following gadolinium administration show minimal enhancement of the lesion. **(c, f)** Diffusion-weighted imaging (DWI, b = 800 s/mm²) and corresponding apparent diffusion coefficient (ADC) maps reveal mildly restricted diffusion.

Ultrasound examination revealed a patchy hypoechoic area with ill-defined margins in segment S2 of the liver (**Figure 1A**). Color Doppler flow imaging (CDFI) demonstrated scattered blood flow signals within and around the lesion (**Figure 1B**). Subsequent contrast-enhanced abdominal CT showed the lesion as a low-density area on the non-contrast phase (**Figure 2A**). During contrast enhancement, the lesion exhibited mild to moderate enhancement, indicative of hypovascularity (**Figure 2B**). In the delayed phase, the enhancement area expanded (**Figure 2C**). The lesion was located in segment II of the left hepatic lobe, measuring approximately 42 mm × 32 mm × 35 mm. Magnetic resonance imaging (MRI) was performed for further characterization. The lesion had poorly defined margins and appeared hyperintense on both T1-weighted (T1WI) and T2-weighted imaging (T2WI) (**Figures 3A, D**). It also demonstrated high signal intensity on diffusion-weighted imaging (DWI) and a slightly decreased signal on the apparent diffusion coefficient (ADC) map, indicating restricted diffusion (**Figures 3C, F**). After administration of Gd-DTPA, the lesion showed minimal enhancement and became nearly indistinct in the delayed phase (**Figures 3D, E**).

Additionally, within the CT scan field of view, an incidental osteolytic lesion with ill-defined margins was detected in the first lumbar vertebra (L1) (**Figure 2F**), without obvious fracture or collapse. CT with soft-tissue windows shows no evidence of paravertebral or epidural soft-tissue involvement(**Figure 2E**).

Considering the hepatic lesion and vertebral involvement, a preliminary diagnosis of malignancy with vertebral metastasis was made. However, due to the nonspecific imaging features, the primary tumor origin could not be determined. Therefore, a biopsy of the liver lesion was scheduled to obtain histopathological confirmation and guide subsequent treatment planning.

**Figure 4:**
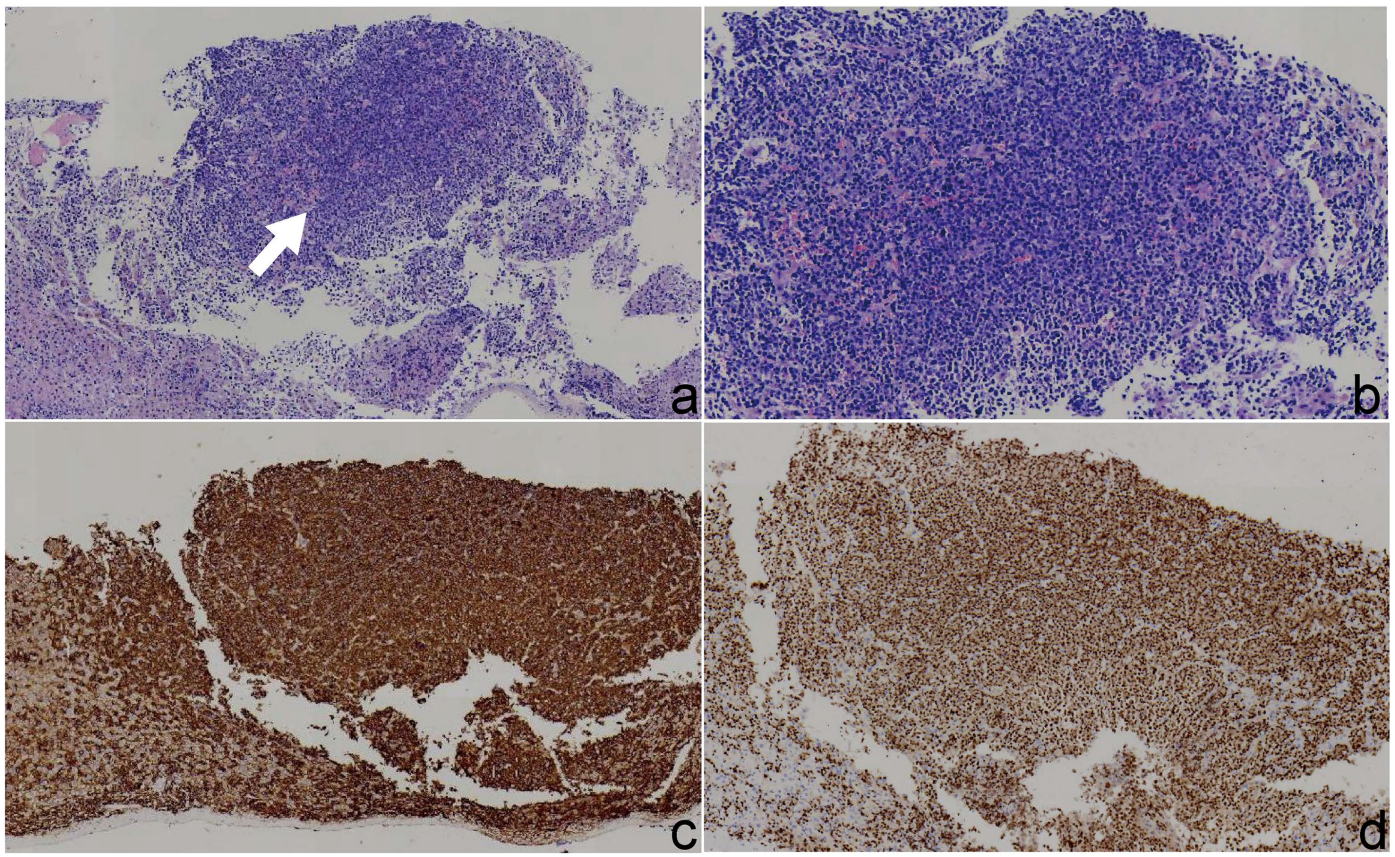
Histopathological and immunohistochemical findings of the liver lesion. **(a)** Hematoxylin and eosin (H&E) staining under low magnification (×4) reveals clusters of atypical plasma cells in the hepatic parenchyma. **(b)** H&E staining under medium magnification (×10) shows diffuse sheet-like infiltration of tumor cells with basophilic cytoplasm. **(c)** Immunohistochemical staining demonstrates strong membranous positivity for CD138 in neoplastic plasma cells. **(d)** Immunohistochemical staining shows nuclear positivity for MUM1 in tumor cells, supporting plasma cell differentiation.

Histological examination revealed diffuse infiltration of atypical plasma cells within the hepatic parenchyma. These cells exhibited abundant cytoplasm, marked nuclear pleomorphism, and occasional mitotic figures. Immunohistochemical staining showed that the tumor cells were positive for CD138, CD38, MUM1 (**Figure 4**), and CD56, with a Ki-67 proliferation index of 70%. Vascular structures stained positive for CD34. A small number of cells also demonstrated positivity for MUC5AC. The tumor cells were negative for CK-P, CK7, CK20, CDX2, Villin, PLAP, MUC2, CA19 - 9, CK19, HepPar-1, Synaptophysin (Syn), Chromogranin A (CgA), OCT4, CD10, CD117, PSA, CD45, CK5/6, and TTF - 1. Vimentin expression was positive. Based on the morphological and immunophenotypic features, a diagnosis of plasmacytoma was suspected. Further hematological testing revealed a markedly elevated serum β_2_-microglobulin level of 7.30 mg/L (reference range: 1 – 3 mg/L), indicating increased plasma cell activity. Consequently, a final diagnosis of multiple myeloma with hepatic involvement was established.

On the fifth day after biopsy, the patient exhibited a marked increase in serum creatinine, reaching 666.3 μmol/L (reference range: 44 – 97 μmol/L), indicative of acute kidney injury, likely postrenal acute renal failure. Decompression via indwelling urinary catheterization proved ineffective. After thorough discussion with the patient and his family, hemodialysis was initiated as supportive treatment. By postoperative day 14, the patient’s renal function and other laboratory parameters had improved compared to previous measurements, showing a declining trend. Owing to the patient’s strong personal preference, he was discharged before commencing systemic therapy for multiple myeloma (e.g., chemotherapy or targeted therapy) and is currently undergoing outpatient follow-up.

## Discussion

Hepatic involvement in multiple myeloma (MM) is an exceedingly rare clinical manifestation. A real-world retrospective study by Beksac et al. involving 226 MM patients with plasmacytomas reported that only approximately 9% of extramedullary myeloma (EM) cases involved the liver (2). Furthermore, Talamo et al. analyzed clinical data from 2,584 MM patients and identified only 11 cases with confirmed hepatic involvement (5), highlighting the extremely low incidence. These findings indicate that hepatic involvement as the initial or primary site in MM is exceedingly uncommon and can easily be misdiagnosed as primary hepatic carcinoma or other metastatic tumors. Hepatic EM generally lacks specific clinical symptoms and laboratory markers; patients often present with mild right upper abdominal discomfort, slight liver function abnormalities, or incidentally discovered hepatic lesions during routine examinations.

Therefore, preoperative diagnosis remains highly challenging. Given that liver biopsy is an invasive procedure and carries certain risks, especially in patients with bleeding tendencies or when lesions are in difficult locations, it is often not feasible as an initial diagnostic approach. Consequently, clinical diagnosis primarily depends on imaging findings for differential assessment.

Extramedullary multiple myeloma (EM) involving the liver may present as focal or multifocal lesions on imaging studies (6, 7). On ultrasonography, the typical manifestation is a well-defined, hypoechoic solid nodule (8). Some reports have described lesions with a “target-like” appearance (9, 10), characterized by central hyperechogenicity and peripheral hypoechogenicity, suggesting internal structural heterogeneity. On non-contrast computed tomography (CT), hepatic EM typically appears as a hypodense nodule or mass within the liver parenchyma, often with poorly defined margins. Contrast-enhanced CT commonly demonstrates marked arterial phase enhancement, with lesions appearing hypodense or isodense during the portal venous and delayed phases (9–12), manifestations similar to those of liver metastases (13–15). Magnetic resonance imaging (MRI) findings include slight hyperintensity or isointensity on T1-weighted images, hyperintensity on T2-weighted images, pronounced hyperintensity on diffusion-weighted imaging (DWI), and hypointensity on apparent diffusion coefficient (ADC) maps, indicating restricted diffusion. On arterial-phase enhanced MRI, lesions often exhibit hyperenhancement (16, 17), followed by isointense signals relative to surrounding liver parenchyma during the portal venous and delayed phases (18, 19).

Magnetic resonance imaging (MRI) further revealed that the lesion was hyperintense on both T1- and T2-weighted images, demonstrated mildly increased signal on diffusion-weighted imaging (DWI), and mildly decreased signal on the apparent diffusion coefficient (ADC) map, indicating only mild diffusion restriction. Contrast-enhanced MRI showed minimal lesion enhancement, which contrasts with previously reported features of extramedullary myeloma (EM), such as hypervascularity and pronounced arterial phase enhancement. Magnetic resonance spectroscopy (MRS) may provide additional metabolic information, such as elevated choline or lipid/lactate peaks, which may help distinguish EM from other hepatic tumors. These findings underscore the considerable variability in the imaging characteristics of hepatic EM, which can closely mimic various primary or metastatic liver tumors, thereby complicating preoperative diagnosis.

^18F-fluorodeoxyglucose positron emission tomography–computed tomography (FDG PET-CT) may provide additional diagnostic value in such scenarios by detecting occult primary malignancies, evaluating systemic disease burden, and identifying other extramedullary sites. In addition, comprehensive skeletal imaging, such as whole-body X-ray, CT, or PET-CT, is recommended to assess for synchronous osseous involvement, which is crucial for staging and management planning. However, in the present case, PET-CT and other skeletal imaging were not performed because our institution does not have PET-CT facilities and the patient declined referral to another center. Given the atypical MRI features, elevated serum β_2_-microglobulin, and clinical suspicion of osseous involvement, direct ultrasound-guided liver biopsy was prioritized to achieve a rapid and definitive diagnosis. Consequently, a definitive diagnosis requires integration of clinical history, comprehensive systemic evaluation, and histopathological confirmation when warranted.

## Conclusion

We report a rare case of hepatic involvement as the initial manifestation of multiple myeloma. The imaging findings—including hypoechoic lesions on ultrasound, low-density masses with mild enhancement on computed tomography (CT), and atypical features on magnetic resonance imaging (MRI)—closely mimicked metastatic liver tumors, resulting in diagnostic confusion. Notably, the MRI enhancement pattern deviated from the hypervascular characteristics commonly reported in extramedullary lesions. This case underscores the diagnostic challenges posed by the heterogeneous imaging presentation of extramedullary myeloma (EM) in the liver and highlights the necessity of including EM in the differential diagnosis of atypical hepatic masses, especially when accompanied by lytic bone lesions or abnormal serum biomarkers. Histopathological confirmation remains essential for definitive diagnosis.

## Data Availability

The original contributions presented in the study are included in the article/supplementary material. Further inquiries can be directed to the corresponding authors.

## References

[1] Bladé J Beksac M Caers J Jurczyszyn A von Lilienfeld-Toal M Moreau P . Extramedullary disease in multiple myeloma: A systematic literature review. Blood Cancer J. (2022) 12:45. doi: 10.1038/s41408-022-00643-3, PMID: 35314675 PMC8938478

[2] Beksac M Seval GC Kanellias N Coriu D Rosiñol L Ozet G . A real world multicenter retrospective study on extramedullary disease from Balkan myeloma study group and Barcelona university: analysis of parameters that improve outcome. Haematologica. (2021) 106:1228. doi: 10.3324/haematol.2020.278272, PMID: 33792227 PMC8018150

[3] Montefusco V Gay F Spada S De Paoli L Di Raimondo F Ribolla R . Outcome of paraosseous extra-medullary disease in newly diagnosed multiple myeloma patients treated with new drugs. Haematologica. (2020) 105:193–200. doi: 10.3324/haematol.2019.219139, PMID: 31221778 PMC6939525

[4] Short KD Rajkumar SV Larson D Buadi F Hayman S Dispenzieri A . Incidence of extramedullary disease in patients with multiple myeloma in the era of novel therapy, and the activity of pomalidomide on extramedullary myeloma. Leukemia. (2011) 25:906–8. doi: 10.1038/leu.2011.29, PMID: 21350560 PMC3736849

[5] Talamo G Cavallo F Zangari M Barlogie B Lee CK Pineda-Roman M . Clinical and biological features of multiple myeloma involving the gastrointestinal system. Haematologica. (2006) 91:964–7., PMID: 16818286

[6] Thomas FB Clausen KP Greenberger NJ . Liver disease in multiple myeloma. Arch Internal Med. (1973) 132:195–202. doi: 10.1001/archinte.1973.03650080039008 4719547

[7] de Vos M Druez P Nicaise M Ngendahayo P Sinapi I Mineur P . Multiple myeloma presenting as hepatic nodular lesion. Acta Clin Belgica. (2012) 67:378–80. doi: 10.2143/acb.67.5.2062696, PMID: 23189550

[8] Tan CH Wang M Fu WJ Vikram R . Nodular extramedullary multiple myeloma: hepatic involvement presenting as hypervascular lesions on Ct. Ann Acad Med Singapore. (2011) 40:329–31. doi: 10.47102/annals-acadmedsg., PMID: 21870026

[9] Cook J Song S Ventimiglia A Luhrs C . Incidental discovery of multiorgan extramedullary plasmacytomas in the setting of newly diagnosed multiple myeloma and delayed hemolytic transfusion reaction. Case Rep Hematol. (2017) 2017:4531858. doi: 10.1155/2017/4531858, PMID: 28761768 PMC5518520

[10] Marcon M Cereser L Girometti R Cataldi P Volpetti S Bazzocchi M . Liver involvement by multiple myeloma presenting as hypervascular focal lesions in a patient with chronic hepatitis B infection. BJR Case Rep. (2016) 2:20150013. doi: 10.1259/bjrcr.20150013, PMID: 30459962 PMC6243346

[11] Kumar K Singh S Nayak J Dhingra G Nath UK . Hepatic plasmacytoma with Del13q14 positive on fluorescent in situ hybridization (Fish) on tissue biopsy. Cureus. (2022) 14:e33197. doi: 10.7759/cureus.33197, PMID: 36726881 PMC9887231

[12] Lee JY Won JH Kim HJ Bae SB Kim CK Kim JH . Solitary extramedullary plasmacytoma of the liver without systemic monoclonal gammopathy. J Korean Med Sci. (2007) 22:754–7. doi: 10.3346/jkms.2007.22.4.754, PMID: 17728524 PMC2693834

[13] Tiu AC Potdar R Arguello-Gerra V Morginstin M . Multiple liver nodules mimicking metastatic disease as initial presentation of multiple myeloma. Case Rep Hematol. (2018) 2018:7954816. doi: 10.1155/2018/7954816, PMID: 29977630 PMC5994292

[14] Erciyestepe M Tiryaki TO Yönal Hindilerden İ Yeğen G Nalçacı M . A case with hepatic involvement mimicking metastatic disease in multiple myeloma. Case Rep Hematol. (2020) 2020:5738319. doi: 10.1155/2020/5738319, PMID: 32733716 PMC7383340

[15] Okamoto Y Ueda Y . Multiple myeloma mimicking liver metastases. Internal Med (Tokyo Japan). (2015) 54:2085–6. doi: 10.2169/internalmedicine.54.4751, PMID: 26278310

[16] Pérez Gil MA Ruiz Recuento J Relanzón Molinero S Martínez Yunta JA . Focal liver lesions in multiple myeloma: ecography, computed tomography, and magnetic resonance findings. A case report. Radiologia. (2006) 48:251–4. doi: 10.1016/s0033-8338(06)73164-2, PMID: 17058655

[17] Kelekis NL Semelka RC Warshauer DM Sallah S . Nodular liver involvement in light chain multiple myeloma: appearance on us and Mri. Clin Imaging. (1997) 21:207–9. doi: 10.1016/s0899-7071(96)00022-8, PMID: 9156311

[18] Papamichail D Hog R Goldschmidt H Dimitrakopoulou-Strauss A . Imaging features of multiple myeloma extramedullary lesions in the liver with 18f-Fdg Pet/Ct, contrast-enhanced Ct and Mri. Diagnost (Basel Switzerland). (2019) 9. doi: 10.3390/diagnostics9040179, PMID: 31703386 PMC6963877

[19] Harada N . Multiple myeloma presenting as nodular hepatic lesions mimicking hepatocellular carcinoma. Int J Hematol. (2024) 119:343–4. doi: 10.1007/s12185-024-03719-x, PMID: 38253959

